# Regulation of Extracellular ATP in Human Erythrocytes Infected with *Plasmodium falciparum*

**DOI:** 10.1371/journal.pone.0096216

**Published:** 2014-05-23

**Authors:** Cora Lilia Alvarez, Julieta Schachter, Ana Acacia de Sá Pinheiro, Leandro de Souza Silva, Sandra Viviana Verstraeten, Pedro Muanis Persechini, Pablo Julio Schwarzbaum

**Affiliations:** 1 Instituto de Química y Fisicoquímica Biológicas (Facultad de Farmacia y Bioquímica), Universidad de Buenos Aires, Buenos Aires, Argentina; 2 Instituto de Biofísica Carlos Chagas Filho, Universidade Federal de Rio de Janeiro, Rio de Janeiro, Brasil; 3 INPeTAm Instituto Nacional de Ciência e Tecnologia em Pesquisa Translacional em Saúde e Ambiente na Reigião Amazônica, Rio de Janeiro, Brasil; Institut national de la santé et de la recherche médicale - Institut Cochin, France

## Abstract

In human erythrocytes (h-RBCs) various stimuli induce increases in [cAMP] that trigger ATP release. The resulting pattern of extracellular ATP accumulation (ATPe kinetics) depends on both ATP release and ATPe degradation by ectoATPase activity. In this study we evaluated ATPe kinetics from primary cultures of h-RBCs infected with *P. falciparum* at various stages of infection (ring, trophozoite and schizont stages). A “3V” mixture containing isoproterenol (β-adrenergic agonist), forskolin (adenylate kinase activator) and papaverine (phosphodiesterase inhibitor) was used to induce cAMP-dependent ATP release. ATPe kinetics of r-RBCs (ring-infected RBCs), t-RBCs (trophozoite-infected RBCs) and s-RBCs (schizont-infected RBCs) showed [ATPe] to peak acutely to a maximum value followed by a slower time dependent decrease. In all intraerythrocytic stages, values of ΔATP_1_ (difference between [ATPe] measured 1 min post-stimulus and basal [ATPe]) increased nonlinearly with parasitemia (from 2 to 12.5%). Under 3V exposure, t-RBCs at parasitemia 94% (t94-RBCs) showed 3.8-fold higher ΔATP_1_ values than in h-RBCs, indicative of upregulated ATP release. Pre-exposure to either 100 µM carbenoxolone, 100 nM mefloquine or 100 µM NPPB reduced ΔATP_1_ to 83–87% for h-RBCs and 63–74% for t94-RBCs. EctoATPase activity, assayed at both low nM concentrations (300–900 nM) and 500 µM exogenous ATPe concentrations increased approx. 400-fold in t94-RBCs, as compared to h-RBCs, while intracellular ATP concentrations of t94-RBCs were 65% that of h-RBCs. In t94-RBCs, production of nitric oxide (NO) was approx. 7-fold higher than in h-RBCs, and was partially inhibited by L-NAME pre-treatment. In media with L-NAME, ΔATP_1_ values were 2.7-times higher in h-RBCs and 4.2-times higher in t94-RBCs, than without L-NAME. Results suggest that *P. falciparum* infection of h-RBCs strongly activates ATP release via Pannexin 1 in these cells. Several processes partially counteracted ATPe accumulation: an upregulated ATPe degradation, an enhanced NO production, and a decreased intracellular ATP concentration.

## Introduction

*Plasmodium falciparum* causes the most severe form of malaria in humans, with ≈200 million cases and ≈ 620.000 deaths in 2012 [1]. Once in the blood, multiplication of the parasite inside erythrocytes (RBCs) is responsible for its severity and mortality associated with the disease [2].

During intraerythrocytic development, infected erythrocytes containing parasites in trophozoite and schizont stages adhere very effectively to the vascular endothelium of capillaries and postcapillary venules. This reduces the vascular lumen and creates a mechanical obstruction to the transit of RBCs [3]. Parasitized RBCs also adhere to uninfected RBCs and other infected RBCs, which further compromises the microvascular blood flow. The situation is even worse during severe malaria, since both parasitized and uninfected RBCs become rigid, a condition which restricts the ability of these cells to flow through capillaries [4], [5].

While these adhesion processes are important determinants of the vascular impairment occurring in infected patients, studies using erythrocytes from healthy individuals (h-RBCs) suggest that the vascular tone might be partially controlled by RBCs themselves. In particular, the vascular caliber of the microcirculation can be modulated by ATP released from erythrocytes [6].

Exposure of h-RBCs to certain physiological and pharmacological stimuli such as hypoxia, β-adrenergic stimulation, prostacyclin analogs, acidity and/or mechanical stress, increases intracellular cAMP with the subsequent stimulation of ATP release [7], [8]. In h-RBCs, receptor-mediated ATP release involves the activation of the heterotrimeric G proteins, Gs or Gi/o [9], [10]. Regarding the Gs pathway of h-RBCs, the binding of various agonists to β-adrenergic receptors stimulate certain isoforms of adenylyl cyclases, with concomitant increases in cAMP levels and the activation of protein kinase A [7], [11].

Moreover, in human and rabbit erythrocytes the direct activation of adenylyl cyclases by forskolin results both in cAMP increase and the stimulation of ATP release [7]. These events are followed by a series of not-well defined intracellular signaling events upstream of ATP release [7], [12].

Human erythrocytes lack intracellular compartments, so that no exocytotic ATP release can occur. Candidate conduits for ATP release of RBCs include anion channels and transporters [13]. Among them, pannexin-1 has been postulated to form hexameric pores that facilitate passive transport of ATP across the plasma membrane [14]–[17]. Pannexin 1-activity can be blocked by carbenoxolone, probenecid or mefloquine [18]–[20], and channel activity consistent with pannexin 1 was recorded in membrane patches excised from h-RBCs [21].

Once in the extracellular milieu, the released extracellular ATP (ATPe) can activate specific P2 receptors (purinergic receptors for di- and trinucleotides) [22] present on adjacent endothelial cells, or it can be hydrolyzed by the ectonucleotidases present on the plasma membrane of RBCs, leukocytes and other vascular cells [23]. In particular, the interaction of ATPe with P2Y receptors on the endothelium stimulates the synthesis of nitric oxide (NO) [6] by the endothelial isoform of the enzyme nitric oxide synthase (eNOS). Upon its diffusion to the extracellular space, NO can interact with, and induce the relaxation of smooth muscle cells surrounding the capillaries of the microvasculature [6], thus causing vasodilation.

Although the evidence above suggests that h-RBCs can act as controllers of the vascular tone, it is presently not known to what extent the impairment of microcirculation in malaria patients is related to alterations in the dynamic balance between ATP release and ATPe degradation from infected RBCs.

We previously demonstrated that a cAMP-activating cocktail (so-called “3V”) containing isoproterenol (a β-adrenergic agonist), forskolin (an activator of adenylyl cyclases) and papaverine (a phosphosdiesterase inhibitor) strongly increased intracellular cAMP concentration and triggered ATP release from h-RBCs [12]. The resulting time-dependent ATPe accumulation (denoted as ATPe kinetics) is governed by the balance between the rates of ATP release (increasing ATPe) and ATPe hydrolysis (decreasing ATPe). However, since ectoATPase activity in h-RBCs is very low (a common feature of RBCs from most mammalian species) [24], the time-dependent changes in [ATPe] are mainly driven by the rate of ATP efflux [12], [25]. Such balance may be altered in infected RBCs as a consequence of metabolic and structural changes induced by the parasite.

In *P. falciparum* infected RBCs the synthesis of cAMP can be acutely enhanced by activation of β2-adrenergic receptor and activation of purinergic adenosine receptor [26], while inhibition of host Gs blocks parasite entry [27].

Parasitized RBCs show a strongly upregulated glycolytic flux, which acts as the sole source of intracellular ATP. Also, additional proteins complexes in the form of knobs are incorporated to the plasma membrane, and thus new ATP efflux conduits and/or ectonucleotidases might appear in infected RBCs.

The effects of *Plasmodium* infection on the rate of ATPe hydrolysis of infected RBCs remain largely unexplored, as well as the implications on ATPe-dependent cell signaling. For example, if the extremely low ectoATPase activity in h-RBCs were upregulated during infection, it would restrain the potential autocrine and paracrine actions of ATPe on infected RBCs. On the other hand, *Plasmodium*-infected RBCs show an increased activity of channels that mediate the flux of a wide variety of organic and inorganic solutes [28]. These new permeability pathways are particularly important for the parasite to get nutrients and release waste products, and to grow [29]. It has been reported that human RBCs have endogenous anion channels, at least one of them being upregulated upon *P. falciparum* infection [30]. Whole cell electrophysiological recordings of trophozoite infected-RBCs evidenced ATP currents sensitive to anion channel inhibitors [31], [32]. This is in line with reports showing that in various cell types ATP might be transported by anionic channels *per se* or as part of a protein complex [19], [25].

One approach to investigate the potential effects of parasite infection on ATPe kinetics of RBCs involves the analysis of the rates of intracellular ATP release and extracellular ATP hydrolysis at different stages of the infection cycle. With this aim, in the present study we investigated the regulation of [ATPe] from uninfected and infected human RBCs stimulated with the cAMP activating cocktail 3V. Particular focus was made on the responses of trophozoite-infected RBCs at parasitemias ranging from 2–12.5% and 94%. Since the pathways for ATP release might change along the infection cycle of the parasite [31], ATPe kinetics was also evaluated in the presence of ATP transport blockers. The comparison of ATPe homeostasis in infected and uninfected RBCs allowed us to speculate on the impact of infection on the control of the vascular caliber by RBCs.

## Materials and Methods

### Reagents

All reagents in this study were of analytical grade. Carbenoxolone (CBX), firefly luciferase (EC 1.13.12.7), sorbitol, forskolin, isoproterenol, papaverine, 5-nitro-2-(3-phenylpropylamino) benzoic acid (NPPB) and Mastoparan 7 (MST7) were purchased from Sigma-Aldrich (St Louis, MO, USA). D-luciferin, Albumax II, RPMI 1640 were obtained from Invitrogen/Molecular Probes Inc. (Brazil) and DAF-FM was obtained from Invitrogen/Molecular Probes Inc. (Argentina). Mefloquine (MFQ) was obtained from BioBlocks QU024-1 Inc (San Diego, CA, USA). L-N-acetyl-methyl-arginine (L-NAME) was purchased from Sigma-Aldrich (St Louis, MO, USA). L-Glutathione reduced (GSH) was kindly provided by Dr M Sterkel (IBQM, UFRJ; Brazil). [^32^Pi]Pi was obtained from the Brazilian Institute of Energetic and Nuclear Research, São Paulo, Brazil. [γ-^32^Pi]ATP was synthesized according to the procedures described by Maia et al. [33].

### Collection and Preparation of Human Erythrocytes

#### 1-Erythrocytes from healthy individuals (h-RBCs)

Samples of h-RBCs were isolated as described before [12]. Erythrocytes were suspended at 45% hematocrit in RPMI medium and stored for 1–7 days at 4°C. Twenty four hours before the experiments, h-RBCs were treated similarly to infected RBCs under culture (see banked h-RBCs in point 2 below). RBCs were suspended at 5% hematocrit in supplemented RPMI medium (RPMI 1640 medium containing 0.5% albumax II, 22 mM glucose, 25 mM HEPES, 0.65 mM hypoxanthine and 50 µg/ml gentamicin) and cultured at 37°C in a 90% N_2_/5% O_2_/5% CO_2_ atmosphere.

Before the experiments, cells were pelleted and resuspended in 300 mosM RBC medium containing (in mM) 137 NaCl, 2.7 KCl, 4.72 Na_2_HPO_4_, 1.50 KH_2_PO_4_, 1.32 CaCl_2_, 1.91 MgSO_4_, 5 glucose, 0.5% bovine serum albumin, pH 7.4 at 25°C.

#### 2-Infected RBCs

Erythrocytic asexual stages of *Plasmodium falciparum* W2 strain, characterized as chloroquine-resistant and mefloquine-sensitive, were maintained in continuous culture in RPMI 1640 medium (Invitrogen, CA, USA) supplemented with 0.5% albumax II (Invitrogen, Brasil), 22 mM glucose, 25 mM HEPES, 0.65 mM hypoxanthine and 50 µg/ml gentamicin [34]. Cultures were maintained at 37°C by routine passage in banked h-RBCs at 5% hematocrit with a final parasitemia of 2–12.5% in a 90% N_2_/5% O_2_/5% CO_2_ atmosphere.

Synchronization to ring-stage was achieved by sorbitol treatment [35]. Infected RBCs were studied at the different stages of parasite development (ring, trophozoite and schizont), and were denoted as r-RBCs (ring-infected RBCs), t-RBCs (trophozoite-infected RBCs) and s-RBCs (schizont-infected RBCs).

Before experiments, thick blood smears were prepared for parasitemia determination by Diff-Quick staining. The percentage of infected cells (parasitemia) in samples was calculated after counting 400 erythrocytes distributed in at least five random microscopic fields.

Aliquots of the culture containing RBCs at a given parasitemia (from 2–12.5%) were pelleted and resuspended as described for h-RBCs.

#### 3-Purification of trophozoites

Suspensions containing synchronized parasite cultures of t-RBCs at 4–7% parasitemia were passed through a magnetic column (MACS LS column, Miltenyi Bioc). This procedure takes advantage of the electromagnetic properties of hemozoin that retains t-RBCs and allows their separation from noninfected cells [36].

Briefly, LS columns were mounted on a high-gradient magnetic cell separator VarioMACS (Miltenyi Biotec), and washed with 5 ml RBC medium before used. A suspension of t-RBCs (parasitemia at 4–7%) was centrifuged 900 ×*g* for 3 min and the pellet was suspended in 2 mL of RBC medium supplemented with 2% bovine serum albumin (BSA) and 2 mM EDTA. The suspension (2×10^8^ t-RBCs mL^-1^) was loaded on and passed through the LS column, and the eluate was reloaded in the same column to optimize t-RBCs retention. The column was washed with RBC medium and removed from the magnetic field. Retained t-RBCs were eluted in RBC medium supplemented with 0.5% BSA. The parasitemia of the final suspension was 94.39±0.03% (N = 15) and subsequently denoted as t94-RBCs.

### Kinetics of Cell Viability

Cell viability was monitored continuously by fluorescence microscopy, as previously described [12], [25]. Briefly, 10^6^ erythrocytes were loaded with BCECF and the retention of the intracellular fluorophore was assessed before and after addition of the pharmacological agents used for the individual experiments. A steep, acute loss of fluorophore was interpreted as cell death. The viability of isolated erythrocytes was assessed every 1 min for 60 min. Results are expressed as the percentage of viable cells.

### Hydrolysis of ATPe

The rate of ATPe hydrolysis was determined by following the accumulation of [^32^P]Pi release from exogenous [γ -^32^P]ATP added to a RBCs suspension of known hematocrit, as described before [12], [25]. Briefly, the reaction was started by the addition of [γ -^32^P]ATP (0.027 Ci/mmol; from 300 to 900 nM) to cell suspensions incubated at 20°C. At different times, 0.2 mL-aliquots of the suspension were withdrawn and centrifuged at 900×g for 30 s, and 0.1 mL of the supernatants were poured onto 0.75 mL of a stop solution containing 4.05 mM (NH_4_)_6_Mo_7_O_24_ and 0.83 mM HClO_4_. The ammonium molybdate solution formed a complex with the released phosphate, which was then extracted with 0.6 mL of isobutyl alcohol. Phases were separated by centrifugation at 1000×g for 5 min, and 0.2 mL-aliquots of the organic phase containing [^32^P]Pi were transferred to vials containing 2 mL of 0.5 M NaOH, and the radioactivity was measured by the Cerenkov effect.

Any hydrolysis of [γ -^32^P]ATP into ADP+[^32^P]Pi in a cell suspension can be defined as ecto-ATPase activity, the time course of which yields a measure of the rate at which one or more ectonucleotidases hydrolyze ATPe. To calculate ectoATPase activity, time dependent levels of P_i_ were fitted to the following equation:
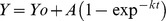
where Y and Y_0_ are the values of [^32^P_i_] at each time (t) and at t = 0, respectively; A represents the maximal value for the increase in Y with time and *k* is a rate coefficient. The parameters of best fit resulting from the regression were used to calculate the initial rate of ectoATPase activity (vi) as *k* × A (*i.e.* the first derivative of Equation 1 evaluated at t = 0). The moles of [^32^P_i_] produced from [γ -^32^P]ATP were calculated from the ATP specific activity [37].

Apparent maximal ectoATPase activity was estimated by measuring ectoATPase activity at 20°C with 0.5 mM ATP. Hyperbolic functions were fitted to experimental results. Initial slopes of these curves were calculated to estimate a pseudo first order constant relating Vi with [ATPe].

### Extracellular ATP and Intracellular ATP Measurements

ATP was measured using firefly luciferase, which catalyzes the oxidation of luciferin in the presence of ATP to produce light [38], [39].

Real-time luminometry measurements of ATPe were carried out with h-RBCs or infected RBCs laid on coverslips that were mounted in the assay chamber of a custom-built luminometer, as previously described [40]. Since luciferase activity at 37°C is only 10% of that observed at 20°C [41], to maintain full luciferase activity, ATP measurements were performed in a cool chamber thermostatized at 20°C. Most measurements were performed using 3×10^6^ cells incubated in 60 µl of RBC medium. Under these conditions the medium has a height of about 104 µm (height at the coverslip bottom of the chamber equals 0). In the case of t94-RBCs, measurements were performed with 0.5–3×10^6^ cells incubated in 60 µl of RBC medium. The time course of light emission was transformed into ATPe concentration *versus* time by means of a built-in calibration curve. For that, increasing concentrations of ATP from 16 to 460 nM were sequentially added to the assay medium from a stock solution of pure ATP dissolved in RBC medium.

Results were expressed as [ATPe] at every time point of a kinetic curve (i.e., ATPe kinetics), with [ATPe] expressed as pmolATP/10^6^ cells or nM/60 µl. Alternatively, increases in [ATPe] were evaluated as the difference between [ATPe] at 1 min post-stimulus and the basal [ATPe], and are indicated as ΔATP_1_.

Total intracellular content of ATP was estimated by permeabilizing cells with digitonin (50 µg/mL) as described before [25]. The released cytosolic ATP was measured by luminometry as described for ATPe. Cytosolic ATP concentration was calculated by considering the total volume occupied by all RBCs present in the chamber, and the relative solvent cell volume in isotonic conditions [42]. Cytosolic volume of t-RBCs was taken from a previous report [43].

In preliminary experiments we found that 100 µM carbenoxolone, 100 nM of mefloquine or 100 µM of NPPB did not affect luciferase activity *in vitro*.

In preliminary experiments using h-RBCs and t-RBCs, we observed that the magnitude of ΔATP_1_ does not depend on ATPe concentration (from 10 to 200 nM).

### Detection of Intracellular Nitric Oxide

Intracellular nitric oxide (NO) was determined using 4-amino-5-methylamino-2′,7′-difluorofluorescein diacetate (DAF-FM DA). This dye emits increased fluorescence after reacting with an active intermediate of NO formed during the spontaneous oxidation of NO to NO_2_^−^ [44].

Before the experiments, h-RBCs and t94-RBCs were incubated for 3 h in supplemented RPMI 1640 medium containing 1.15 mM L-arginine, either in the absence or presence of 2 mM of the NOS inhibitor L-N-acetyl-methyl-arginine (L-NAME). Cells were centrifuged for 3 min at 900×g. Suspensions of h-RBCs and t94-RBCs (10^7^ cells/(ml) in RBC medium) were incubated for 60 min at 20°C in the presence of 5 µM DAF-FM DA, and washed three times with RBC medium to remove non-incorporated probe. Fluorescence was recorded at 510–550 nm (λexcitation: 490 nm) in a SpectraMax M5 fluorescent microplate reader using a final sample volume of 0.1 mL. Fluorescence intensity was monitored continuously in the absence of treatments (basal trace) or in the presence of 3V. At the end of the experiment, 1 mM of freshly prepared S-nitrosoglutathione (GSNO) [45] was added as a positive control of cell loading with the probe.

### Extracellular Nitrite Content

Nitrite release from h-RBCs and t-RBCs at different parasitemias was used to estimate NO production.

Nitrite content in the medium was determined colorimetrically using Griess reagent [46]. Cells were incubated for 24 hs at 37°C in supplemented RPMI medium. At the end of incubation, aliquots of cell suspensions were withdrawn, centrifuged at 900×*g* for 3 min, and 50 µl of the supernatant was mixed with an equal volume of Griess reagent (1% sulfanilamide and 0.1% N-(1-naphthyl)-ethylenediamine in 5% phosphoric acid). Nitrite concentrations were determined at 550 nm by comparison with standard solutions run in parallel and containing sodium nitrite in RPMI 1640 medium. Each experiment was performed in duplicate and repeated at least three times.

### Ethics Statement

All procedures conformed to the Declaration of Helsinki. Collection of human blood samples for this study was conducted according to the protocols approved by the Research Ethics Committee of the Hospital Universitário Clementino Fraga Filho from Federal University of Rio de Janeiro (Permit Number 074/10).

All healthy donors provided written informed consent for the collection of samples and subsequent use. The use of this material follows long-standing protocols and has not been associated with any adverse or other unforeseen events and no data of relevance to specific patients has been generated.

### Treatments

ATP release was induced with the 3V mixture, which contained 10 µM isoproterenol, 30 µM forskolin and 100 µM papaverin [12]. Carbenoxolone 100 µM, 100 nM mefloquine or 100 µM NPPB were used as blockers of Pannexin 1.

In experiments shown in Fig. S4, ATP release of h- and t94-RBCs was induced by exposing cells to 10 µM of the peptide mastoparan 7 (MST7).

### Data Analysis

Statistical significance was determined using Kruskal-Wallis Test followed by a Dunn’s Multiple Comparison Test. A p value <0.05 was considered significant. Numbers of determinations (n) from independent preparations (N) are indicated. For viability experiments showed in Fig. S2, 10^6^ cells from 3–4 independent preparations were used.

Spearman rank correlation was calculated to analyze data of ΔATP_1_ as a function of parasitemia (%) for infected-RBCs.

The kinetics of intracellular NO production in h-RBCs and t-RBCS (in the absence and presence of L-NAME) was compared using the slope comparison built-in test from GraphPad Prism version 5.00 for Windows, GraphPad Software (San Diego, CA, USA).

## Results

### ATPe Kinetics of 3V-exposed h-RBCs

**Figure 1:**
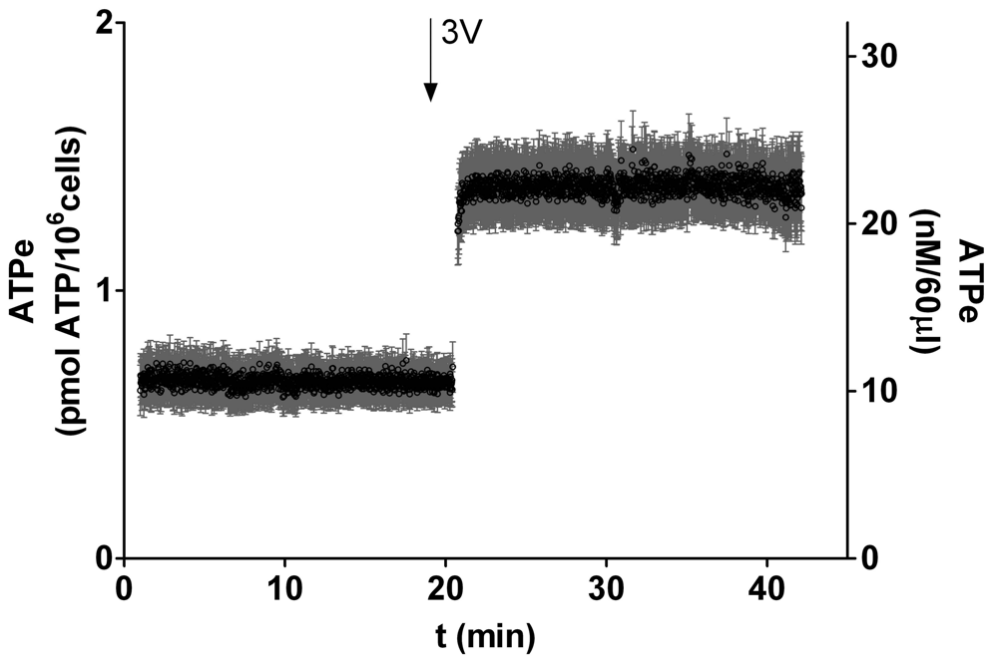
Kinetics of ATPe from cAMP-stimulated human erythrocytes (h-RBCs). The time course of ATPe concentration ([ATPe]) from h-RBCs was quantified by real-time luminometry, as described in Materials and Methods. In the time indicated by the arrow, cells were exposed to “3V”, a cAMP activating cocktail containing 10 mM isoproterenol, 30 mM forskolin and 100 mM papaverine. Levels of ATPe were expressed both as pmol ATP/(10^6^ cells) (left axis) or as ATPe concentration (nM) with 10^6^ cells in 60 µl assay volume (right axis). Data represent mean values ± SEM from N = 14 independent preparations.

In Fig. 1, a quantification of the time dependent accumulation of ATPe from h-RBCs is shown, and denoted as *ATPe kinetics*, which depends on both the rates of ATP release (promoting an increase in [ATPe]) and ATPe hydrolysis (promoting a decrease in [ATPe]).

In nonstimulated h-RBCs, [ATPe] remained steady at 0.64±0.07 pmoles/(10^6^ cells). ATP release was next stimulated by adding the cAMP-activating cocktail 3V [12]. A fast 2 fold-increase in [ATPe] was observed after 3V addition, with [ATPe] reaching a maximal value of 1.35±0.12 pmoles/(10^6^ cells), which remained constant up to 50 min of incubation (Fig. 1). The fast relative increase in [ATPe], denoted as ΔATP_1_, was estimated as the difference between [ATPe] measured 1 min post-stimulus and the basal [ATPe] measured prior to cells stimulation. ΔATP_1_ amounted to 0.71±0.09 pmoles/(10^6^ cells).

### ATPe Kinetics of Infected RBCs Exposed to 3V

We evaluated ATPe kinetics and ΔATP_1_ of infected RBCs at different stages of parasite development.

**Figure 2:**
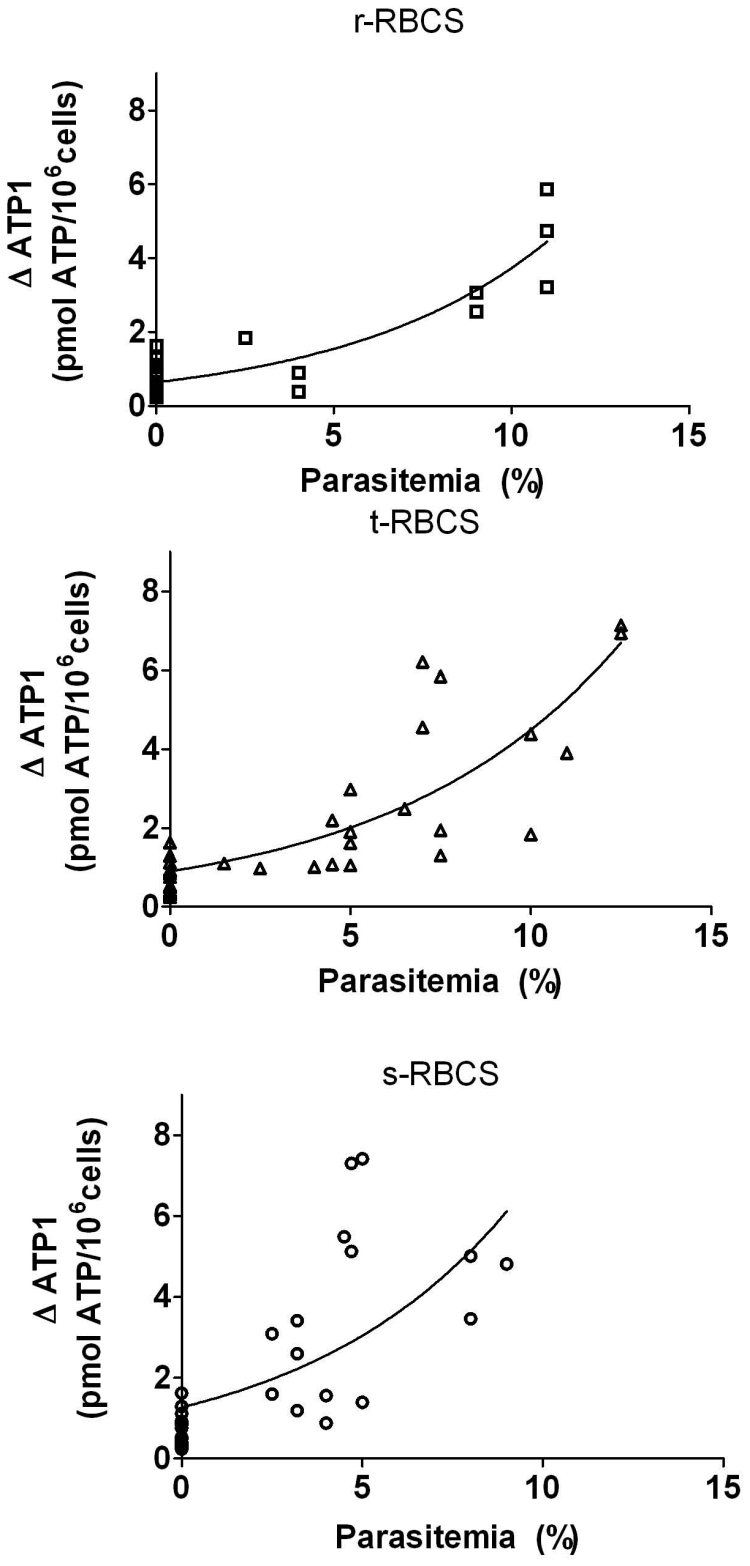
3V-dependent increase of [ATPe] of *P. falciparum* infected RBCs. Values are expressed as ΔATP_1_, i.e., the difference between [ATPe] at 1 min post-stimulus and basal [ATPe]. ΔATP_1_ was plotted as a function of parasitemia (%) for r-RBCs (ring-infected erythrocytes; N = 8, n = 5), t-RBCs (trophozoite-infected erythrocytes; N = 19, n = 15) and s-RBCs (schizont-infected erythrocytes; N = 12, n = 15). N = independent preparations, n = replicates. The continuous lines represent positive parabolic functions fitted to experimental data. Spearman correlation coefficients were 0.6212, 0.8438 and 0.7946 to r, t and s-RBCs, respectively.

In r-RBCs (ring-infected RBCs), t-RBCs (trophozoite-infected RBCs) and s-RBCs (schizont-infected RBCs) ΔATP_1_ increased in a nonlinear fashion with parasitemia (Fig. 2). The higher dispersion of data using s-RBCs (as compared to r- and t-RBCs) could be due to the highly leaky membranes of these cells [47].

**Figure 3:**
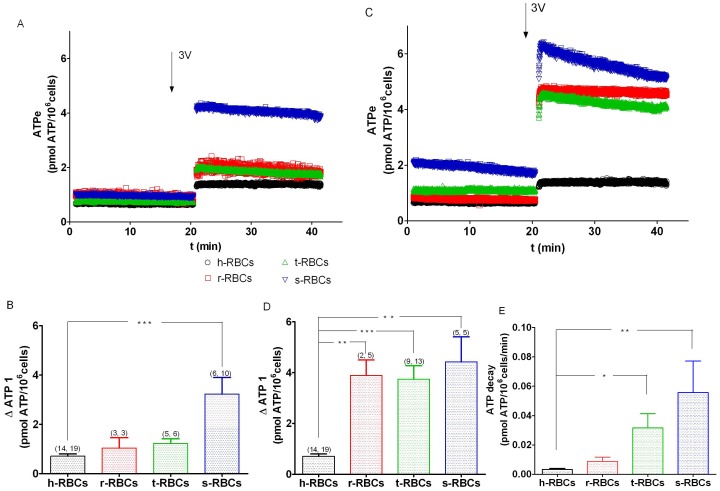
3V-dependent ATP kinetics of *P. falciparum* infected RBCs. A, C. Time course of ATPe concentration (pmol/10^6^ cells) for r-RBCs (ring-infected erythrocytes), t-RBCs (trophozoite-infected erythrocytes) and s-RBCs (schizont-infected erythrocytes) at low parasitemia (<5%, A) and high parasitemia (5–12.5%, C). In the time indicated by the arrow, cells were exposed to “3V”, a cAMP activating cocktail containing 10 mM isoproterenol, 30 mM forskolin and 100 mM papaverine. For a comparison, similar experiments with h-RBCs are shown. B, D. 3V-dependent increases of [ATPe] calculated from A and C. Values are expressed as ΔATP_1_ for low parasitemia (B) and high parasitemia (D). Results are means ± SEM. (*p<0.05, ***p<0.001). (N, n), with N = independent preparations, n = replicates. E. Initial rate of [ATPe] decay (pmol/10^6^ cells/min) taken from data of C.

To evaluate ATPe kinetics of infected RBCs, data were grouped into low (<5%) and high (5–12.5%) parasitemia (Fig. 3). At low parasitemia, slight but not significant changes in ATPe kinetics and ΔATP_1_ were observed in r- and t-RBCs, whereas s-RBCs exhibited a strong increase in time dependent [ATPe] accumulation, yielding a 4.5-fold increase in ΔATP_1_ with respect to noninfected h-RBCs (Fig. 3A–B).

At high parasitemia, on the other hand, [ATPe] levels were higher at all times post-stimulus with respect to h-RBCs (Fig. 3C). ATPe kinetics of infected RBCs showed [ATPe] to peak acutely to a maximum value followed by a time dependent decrease. ΔATP_1_ values in r-RBCs, t-RBCs and s-RBCs were, respectively, 5.5-, 5.3- and 6.2-fold higher than in h-RBCs (Fig. 3D). The rates of [ATPe] decay, an indirect measure of ectoATPase activities, increased with the progress of the cycle (Fig. 3E).

The consequence of parasite infection on ATPe regulation was further evaluated by studying [ATPe] kinetics and ectoATPase activity of t-RBCs at parasitemias ≈5 (t5-RBCs) or ≈94% (t94-RBCs) and h-RBCs.

### Conduits of ATP Release in an Enriched Population of t-RBCs (t94-RBCs)

3V-dependent ATPe kinetics was studied in t94-RBCs in the absence and presence of three pannexin1 inhibitors: carbenoxolone (CBX), mefloquine (MFQ) or 5-nitro-2-(3-phenylpropylamino) benzoic acid (NPPB) [20]. Results were compared with similar experiments using h-RBCs.

**Figure 4:**
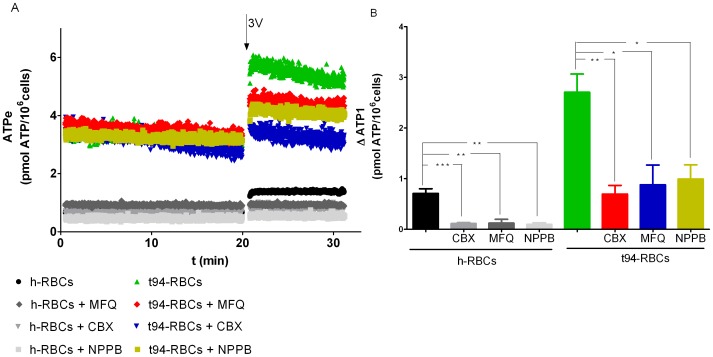
Effect of Pannexin 1 inhibitors on [ATPe] kinetics of a highly enriched population of trophozoite infected erythrocytes. A. The time course of [ATPe] (pmol/10^6^ cells) was assessed for trophozoite-infected erythrocytes at 94% parasitemia (denoted as t94-RBCs) in the absence and presence of 100 µM carbenoxolone (CBX), 100 nM mefloquine (MFQ), or 100 µM of 5-nitro-2-(3-phenylpropylamino) benzoic acid (NPPB), inhibitors of Pannexin 1. Exposure to 3V is indicated by the arrow. In some experiments, prior to 3V exposure cells were pre-incubated 10 min with either CBX, MFQ or NPPB. For a comparison, similar experiments with noninfected RBCs (h-RBCs) are shown. t94-RBCs (N = 14, n = 19), t94-RBCs +CBX (N = 6, n = 7), t94-RBCs+MFQ (N = 4, n = 4), t94-RBCs+NPPB (N = 4, n = 4), h-RBCs (N = 15; n = 19), h-RBCs+CBX (N = 6, n = 9), h-RBCs+MFQ (N = 4, n = 4), h-RBCs+NPPB (N = 3, n = 3). N = independent preparations, n = replicates. B. 3V-dependent increase of [ATPe] calculated from A. Values are expressed as ΔATP_1_, i.e., the difference between [ATPe] at 1 min post-stimulus and basal [ATPe]. Results are means ± SEM. (*p<0.05, ***p<0.001).

At all times assessed, ATPe kinetics in t94-RBCs displayed much higher ATPe concentrations than in h-RBCs (Fig. 4A). Unlike results obtained with h-RBCs, both the basal and post-stimulus traces showed a continuous time-dependent decrease of [ATPe], suggesting a significant ectoATPase activity. A qualitatively similar ATPe kinetics was also observed in mice t-RBCs infected with *P. chabaudi* (Fig. S3A). Values of ΔATP_1_ were 3.8- fold higher for t94-RBCs than for h-RBCs (Fig. 4B).

Cells pre-incubation with 100 µM CBX, 100 nM MFQ or 100 µM NPPB reduced ΔATP_1_ by 63–74% in t94-RBCs, and 83–87% in h-RBCs (Fig. 4B).

In a few experiments (Fig. S4) h- and t94-RBCs were exposed to MST7. At all times ATPe concentrations were much higher in t94- than in h-RBCs. The resulting ΔATP1 values were 5.73±1.19 (t94-RBCs) and 0.89±0.43 (h-RBCs) pmoles/(10^6^ cells). Values of ΔATP_1_ were 6.4- fold higher for t94-RBCs than for h-RBCs (Fig. S4B).

### EctoATPase Activity in h- and t-RBCs

**Figure 5:**
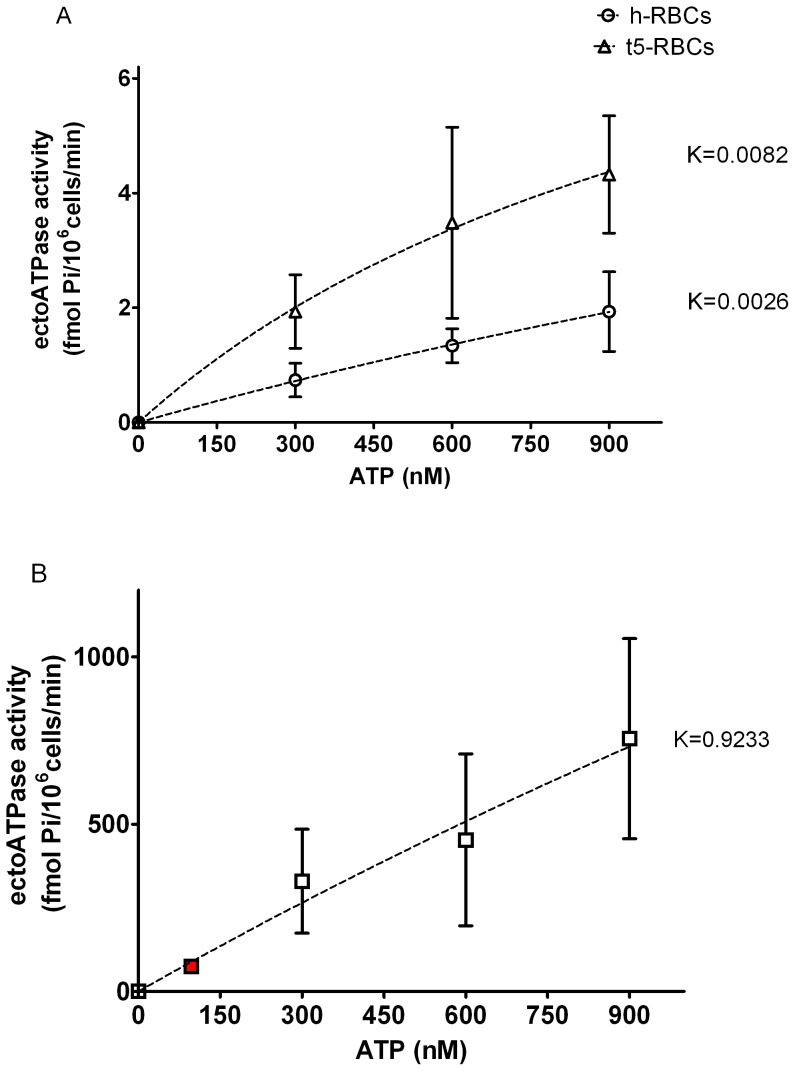
EctoATPase activity as a function of exogenous [ATP] of h-RBCs and t-RBCs. Each symbol represents ectoATPase activity calculated as the initial rate of Pi accumulation ([^32^P]Pi) released from exogenous [γ-^32^P]ATP (300, 600, and 900 nM). Experiments were run using suspensions of: A. Noninfected RBCs (h-RBCs, N = 5, n = 5) and trophozoite-infected RBCs at 5% parasitemia (t5-RBCs, N = 4, n = 4); B. Trophozoite infected RBCs at 94% parasitemia (t94-RBCs, N = 6, n = 6). The dotted lines represent fitting of hyperbolic functions to experimental data. Initial slopes of these curves were calculated to estimate a pseudo first order constant (K) relating ectoATPase activity with [ATPe]. Values of K are given in brackets. Results are means ± SEM. The red square in 5B represents an estimate of ectoATPase activity by real time luminometry. It was calculated from post-stimulus ATPe decay kinetics of t94-RBCs from Fig. 4A.

The dependence of ectoATPase activity on [ATPe] was studied using suspensions of intact h-, t5- and t94-RBCs (Fig. 5). Except for the red symbol indicated in Fig. 5B, each experimental point was determined from the time course of [^32^P]Pi accumulation released from [γ-^32^P]ATP (300–900 nM).

In t-RBCs, ectoATPase activity increased with ATP concentration in the reaction media (Fig. 5). Initial slopes of the substrate curves were 3.2- (t5-RBCs) and 360-fold (t94-RBCs) higher than in h-RBCs.

EctoATPase activity could also be estimated by the luminiscence experiments of Fig. 4A, where [ATPe] of 3V exposed t94-RBCs increased to a maximum followed by a nonlinear decay (Fig. 4A). Accordingly, the result indicated by a red symbol in Fig. 5B is an estimation of the ectoATPase activity calculated from that post-stimulus decay rate of [ATPe]. This point extrapolates well to the ectoATPase activities calculated by the radioactive method.

**Figure 6:**
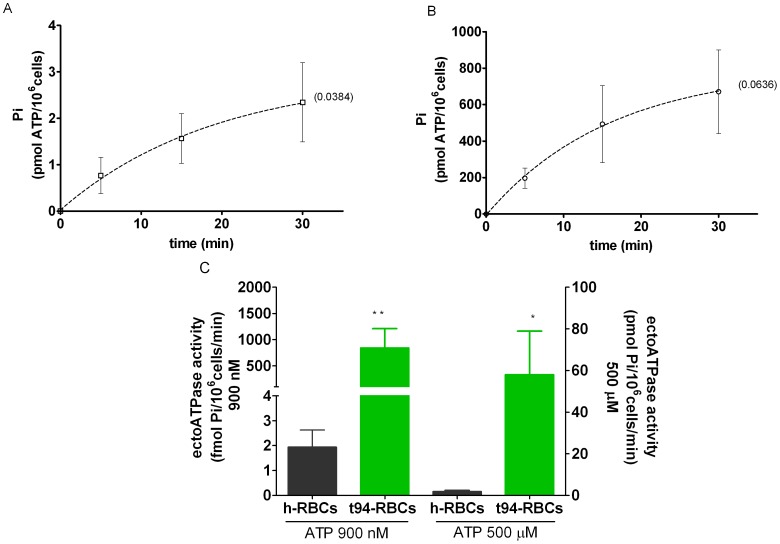
Apparent maximal ectoATPase activities of h-RBCs and t94-RBCs. A, B. Rates of Pi accumulation ([^32^P]Pi) released from exogenous 500 µM [γ-^32^P]ATP, using suspensions of noninfected RBCs (h-RBCs, N = 4, n = 4) and trophozoite-infected RBCs at 94% parasitemia (t94-RBCs; N = 3, n = 4). The dotted lines represent the fitting of exponential functions to experimental data, with values of the corresponding rate constant (k) given in brackets. Values of best fit were used to calculate apparent maximal ectoATPase activities as described in Materials and Methods. C. Apparent maximal ectoATPase activities at 500 µM ATP were determined from exponential fits of A and B. For a comparison, ectoATPase activities of h- and t94-RBCs at 900 nM ATP, taken from Fig. 5, are shown. Significant differences are indicated (*, p<0,05, **, p<0,01). Results are means ± SEM.

Finally, in h- and t94-RBCs, the apparent maximal ectoATPase activities were estimated from the time course of [^32^P]Pi accumulation released using 500 µM concentration of [γ-^32^P]ATP (Fig. 6A–B). Apparent maximal ectoATPase activities amounted to 0.15±0.05 pmol Pi/(10^6^ cells min) in h-RBCs and 58±21 pmol Pi/(10^6^ cells min) in t94-RBCs (Fig. 6C). The ectoATPase activity of t94-RBCs was 442-fold and 380-fold higher than those of h-RBCs at 900 nM and 500 µM ATP concentrations, respectively (Fig. 6C).

### NO Production and ATP Release

**Figure 7:**
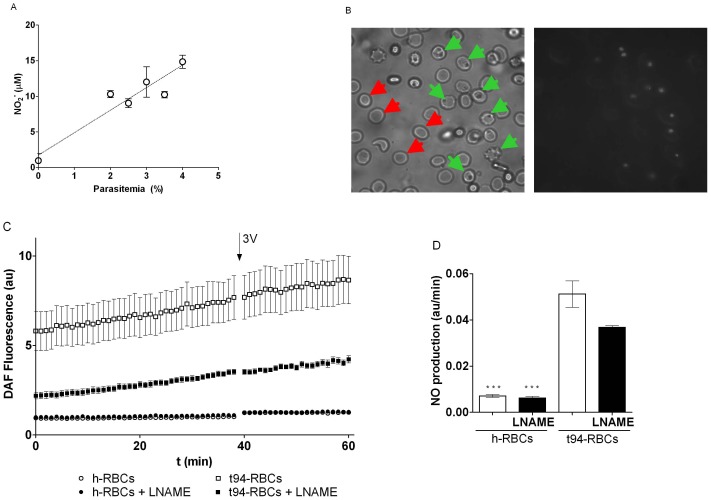
Nitric oxide production. A. Extracellular nitrite (NO_2_^−^) content as a function of parasitemia for trophozoite-infected RBCs (t-RBCs, 0–4% parasitemia) incubated for 24 hs in supplemented RPMI medium. Cells were cultured in an atmosphere of 90% N_2_/5% O_2_/5% CO_2_ at 37°C containing 1.15 mM L-arginine. B. Microphotographies from DAF-2 diacetate loaded RBCs under bright light (left) and fluorescence illumination (right). Green arrows show trophozoite-infected cells, and red arrows indicate noninfected RBCs. Total magnification 1000X. C. DAF-2 diacetate fluorescence intensities of cell suspensions as a function of time, determined by fluorometry. Experiments were run using h- and t94-RBCs. Before the experiments, cells were pre-incubated 3 hs in the absence and presence of 2 mM L-NAME. D. NO production was estimated from the slopes of linear fits to data shown in C. Results (au/min) are means ± SEM with N = 3, n = 6–12. Significant differences are indicated (*, p<0,05, **, p<0,01).

In t-RBCs incubated for 24 h in supplemented RPMI medium, the extracellular concentration of nitrite, indicative of NO production, increased linearly (r = 0.84) with cell parasitemia within the 2–4% range (Fig. 7A). This finding indicates that NO production increases during *P. falciparum* infection.

Next, a series of experiments were made to test a possible relationship between intracellular NO production and ATP release.

T5-RBCs and h-RBC were pre-incubated in supplemented RPMI medium for 3 h in the absence or presence of 2 mM L-NAME. Cells were subsequently purified to obtain t94-RBCs and divided in two aliquots to assess simultaneously the intracellular NO production and ATPe kinetics.

Micrographs of DAF fluorescence showed that NO synthesis occurred at the site where the parasites were located (Fig. 7B). Next, the kinetics of NO generation was monitored in DAF-FM loaded cells by fluorescence quantification (Fig. 7C), with the slopes of each curve representing intracellular NO production for the different experimental conditions (Fig. 7D). In h-RBCs NO production was minimal, as evidenced from the slow increase in DAF fluorescence over the period assessed, and was not inhibited by L-NAME pre-treatment (Fig. 7C). On the other hand, in t94-RBCs, a significant NO production was evidenced, which was approximately 7.2-times higher than the measured in h-RBCs and was inhibited to 72% by L-NAME pre-treatment (Fig. 7D). Supporting that, at the beginning of the measurements, DAF fluorescence in t94-RBCs with L-NAME represented only 38% of that found in cells incubated in the absence of L-NAME (Fig. 7C).

To verify that the observed differences in DAF-FM oxidation between cell populations were not due to differential cell loading with the probe, at the end of experiments samples were added with 1 mM of NO donor GSNO to achieve maximal and equal NO generation. Under these conditions, DAF fluorescence increased ∼150 times until reaching a plateau after 20 min of GSNO addition (data not shown).

**Figure 8:**
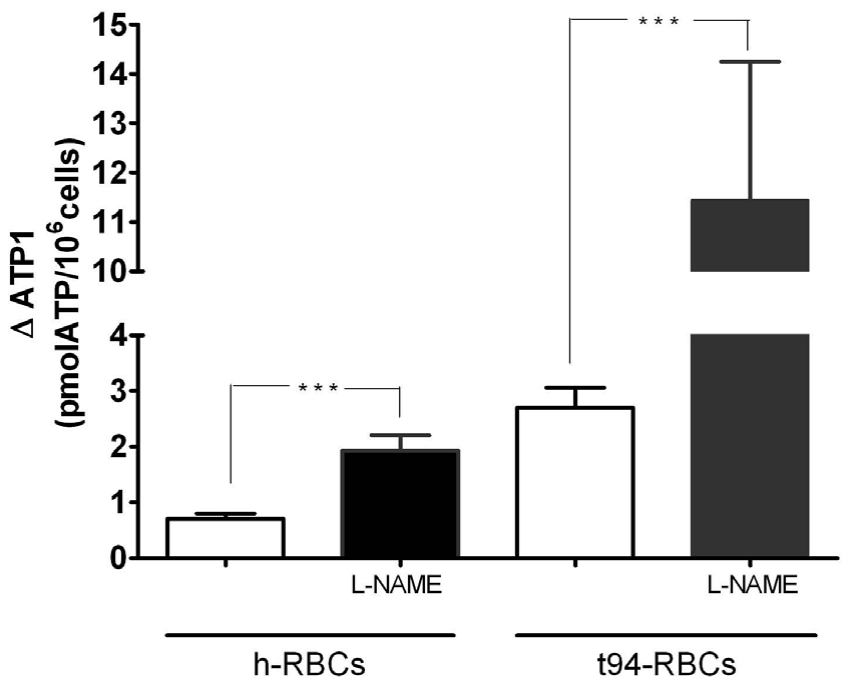
Effect of L-NAME on 3V-dependent increase of [ATPe] in h-RBCs and t94-RBCs. Values are expressed as ΔATP_1_, i.e., the difference between [ATPe] at 1 min post-stimulus and basal [ATPe]. Before the experiments, cells were pre-incubated 3 hs in the absence and presence of 2 mM L-NAME. A. ΔATP_1_ values for noninfected and trophozoite-infected RBCs (h- and t94-RBCs) in the absence and presence of L-NAME. Results are means ± SEM with N = 4 and n = 4. (*p<0.05, ***p<0.001).

The role of NO on 3V-dependent ATP release was estimated by calculating ΔATP_1_ values of h- and t94-RBCs exposed to 3V in a media with or without 2 mM L-NAME, and in the absence or presence of CBX (100 µM). In the presence of L-NAME, ΔATP_1_ values were 2.7-times higher in h-RBCs and 4.2-times higher in t94-RBCs than their corresponding values measured in the absence of L-NAME (Fig. 8). When pre-incubated in the presence of CBX, t94-RBCs showed similar ΔATP_1_ values regardless the presence of L-NAME (0.82±0.14 and 0.70±0.17 for cells with and without L-NAME, respectively).

**Figure 9:**
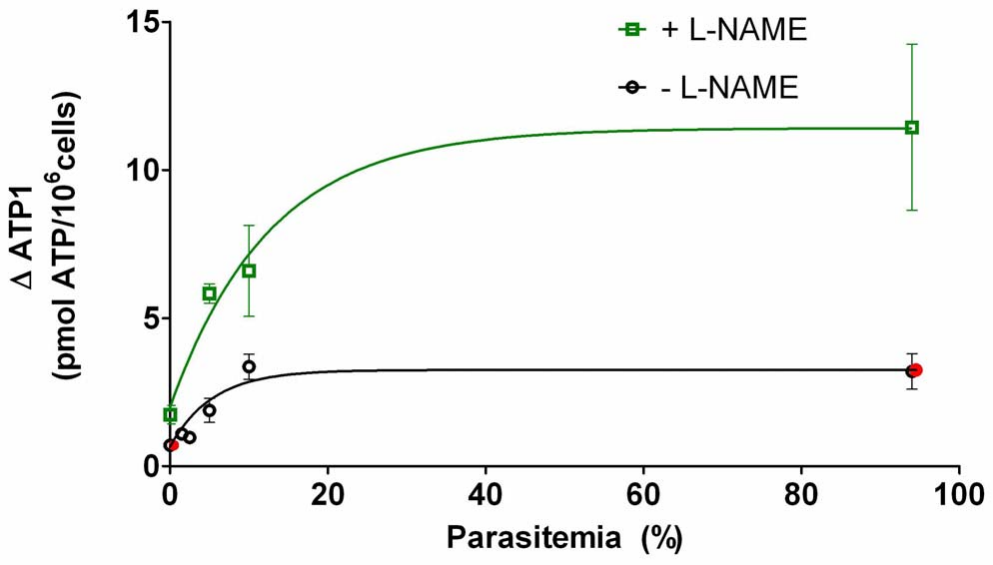
3V-dependent increase of [ATPe] of trophozoite-infected RBCs. Values of ΔATP_1_ as a function of parasitemia (5–12.5%) for trophozoite-infected RBCs (N = 4, n = 4–5). Prior to experiments, cells were pre-incubated 3 hours in the absence (black circles) or presence (green squares) of 2 mM L-NAME. Hyperbolic functions were fitted to experimental data. Results are means ± SEM with N = 3 and n = 5–10. N = independent preparations, n = replicates. The red symbols illustrate an estimate of ΔATP_1_ under a hypothetical situation where ectoATPase activity is blocked. It was calculated by: 1- estimating the concentration of ATPe hydrolyzed during the first minute post-stimulus (using results of Fig. 5); 2- adding that value to the experimentally obtained ΔATP_1_.

ΔATP_1_ was also evaluated for t-RBCs at different parasitemias, except that cells were not only pre-incubated but also assayed in the presence of 2 mM L-NAME (Fig. 9). Both, in the presence and absence of L-NAME, ΔATP_1_ increased hyperbolically with the parasitemia, with values being significantly higher in the presence of L-NAME. Red symbols represent an estimation of ΔATP_1_ in a hypothetical situation where ectoATPase activity was blocked.

### Intracellular ATP Concentration

The intracellular ATP content of t94-RBCs and h-RBCs was determined in nonstimulated cells. By considering the cytosolic volume of these cells [43], the intracellular ATP concentration could be derived. Accordingly, ATPi concentration was 1.82±0.08 mM in h-RBCs, and 1.18±0.11 mM in t94-RBCs, significantly smaller (Fig. S1A).

## Discussion

Infection by *P. falciparum* can cause multiple vital organ dysfunctions despite the use of new generation antimalarial drugs and appropriate clinical care [48]. Among the possible mechanisms contributing to the disease are those affecting the flow in the microcirculation because of the capacity of t-RBCs and s-RBCs to adhere to the vascular endothelium and reduce the vascular lumen.

In addition to this canonical process connecting cytoadherence with impaired microcirculatory flow, other mechanisms affecting blood circulation may coexist. In this study we tested whether *P. falciparum* infected RBCs exhibited an altered ATPe homeostasis that would allow these cells to participate more intensely in the regulation of the vascular caliber.

### ATPe Kinetics

As a first approach we exposed infected RBCs to “3V” and measured the time- dependent accumulation of [ATPe] in all intraerythrocytic stages of *Plasmodium*. In h-RBCs this treatment led to an acute increase of cAMP, which triggered the release of ATP [12]. Interestingly, infected RBCs released more ATP upon stimulation with 3V (estimated as ΔATP_1_) than h-RBCs. The response of infected cells to 3V occurred at all stages of cell infection and was influenced by the relative proportion of infected cells in the samples (an “*in vitro*” parasitemia).

To analyze ATPe kinetics under conditions that reproduce those observed in malaria patients, experiments were performed either at low (<5%) or high (5–12.5%) degree of parasitemia. The low parasitemia group represented the average condition normally found in malaria patients, where parasitemia in peripheral blood is generally below 4% [49]. However, *in vivo* a much higher percentage of infected erythrocytes can be found at specific local points. For example, during rosetting, t-RBCs and s-RBCs are able to bind to two or more uninfected erythrocytes and thus raise locally the effective parasitemia up to 30% [50].

Two important features of ATPe homeostasis arise from these observations. First, at high parasitemia, the increase in ΔATP_1_ in infected cells was 5–6 times higher than that measured in normal h-RBCs, indicating a strong activation of ATP efflux that occurred independently of parasite developmental stage. Second, [ATPe] kinetics from h-RBCs and infected RBCs was different. Under 3V stimulation, [ATPe] of h-RBCs increased rapidly to a saturation value (Fig. 1) that remained constant thereafter, while in infected RBCs [ATPe] peaked acutely to a maximum value and progressively decreased over time (Fig. 4). The finding that in infected RBCs, both prior and shortly after 3V exposure, the levels of [ATPe] decreased steadily suggests an upregulation of ectoATPase activity in these cells, a feature that was also observed in mice t-RBCs infected with *P. chabaudi* (Fig. S3). This and other aspects of [ATPe] kinetics were further evaluated using h-RBCs and trophozoite-infected RBCs at parasitemias 5% (t5-RBCs) or 94% (t94-RBCs). Particular attention was given to analyzing the responses of t94-RBCs. This constitutes an almost pure population of trophozoite infected RBCs, far from the parasitemia levels usually found *in vivo*, but nevertheless important to investigate the properties of t-RBCs in the absence of interactions with noninfected RBCs.

### EctoATPase Activity

EctoATPase activity can be estimated from the capacity of intact cells to hydrolyze exogenous ATP. The concentrations of ATP chosen for this assessment were selected from the physiological ATP values found in human plasma, which span the nanomolar range of concentrations [41]. Both in intact h-RBCs and t-RBCs, ectoATPase activity increased with the increase of exogenous ATP concentration. In t-5 and t-94 RBCs, the initial slopes of the substrate curves (calculated as the ratio between ectoATPase activity and the concentration of added ATP) were 3.2- and 360-fold higher in t5- and t94-RBCs than in h-RBCs, respectively.

How can this elevated ectoATPase activity be achieved during *P. falciparum* infection? Human erythrocytes lack organelles and nucleus and thus the synthesis of new proteins will be restricted to the translation of pre-existing mRNA. However, the observed higher ectoATPase activities of t-RBCs at low –but physiological- nanomolar ATPe concentrations (Fig. 5), as compared to those of h-RBCs, could be due to an increased expression of one or more ectonucleotidases produced by the parasite.

In several cell types ATPe hydrolysis is facilitated by ecto-nucleoside triphosphate diphosphohydrolases (ENTPDase), a family of membrane-bound ectonucleotidases with broad specificity for di- and triphosphate nucleosides [51], [52]. Although it has been reported that *P. falciparum* genome encodes for a single ENTPDase [53] no previous account of ATPe hydrolysis from intact infected RBCs has been reported. If the plasma membrane from infected cells contains increased ectoATPase activity levels as a consequence of parasite induced expression of ectonucleotidases, concomitant changes in the maximal rate of ATPe hydrolysis should be observed. To verify this hypothesis, ectoATPase activity of intact cells was evaluated at 500 µM ATP, which based on the reported K_0.5_ATP of most ectonucleotidases [54], should approach apparent maximal ectoATPase activity. Under this condition, the rate of ATPe hydrolysis for t94-RBCs was 380-fold higher than that of h-RBCs. In addition, the relative increase in ectoATPase activity of t94- respect to h-RBCs was similar at two extreme ATPe concentrations such as 900 nM (442-fold increase) and 500 µM (380-fold increase), a finding that supports the hypothesis that infected cells express higher levels of functional ectonucleotidases than noninfected cells.

Interestingly, the experimentally observed ectoATPase activities in the physiological nanomolar range lie well below the maximal capacities of the implicated ectonucleotidases. Thus we predict that, as stimulated h-RBCs and t-RBCs experience increased ATP concentrations at the cell surface, ectoATPase activity will be activated accordingly, following its substrate curve. The fact that plasma [ATPe] from malaria patients is elevated [55] is in agreement with the 100-fold increase in [ATPe] found *in vitro* in t-RBCs cultures (Fig. S1B). Therefore, in addition to the higher ectoATPase activity found in t-RBCs, *in vivo* this enzyme(s) will function at much higher ATPe concentrations and thus at higher ATPe hydrolysis rates than in h-RBCs, which are physiologically exposed to lower ATPe concentrations.

As already mentioned, ectoATPase activity of h-RBCs is very low, and does not contribute significantly to [ATPe] kinetics. In t-RBCs, on the other hand, the strongly elevated ectoATPase activity suggests that ATPe degradation by ectoATPase activity should be able to alter ATPe kinetics. This explains the decay of [ATPe] of t94-RBCs, a feature not observed in h-RBCs (Fig. 4A). On the other hand, during the first minute post-stimulus, where ATP efflux is strongly activated, ectoATPase activity contributes little to ATPe kinetics (as shown in Fig. 1).

### Conduits of ATP Release

Considerations presented above indicate that during the first minute post-stimulus, [ATPe] increases (estimated as ΔATP_1_) are almost exclusively driven by ATP efflux, but do h-RBCs and infected RBCs use similar ATP conduits? Conductive or transport mechanisms were reported to mediate ATP release. In particular, pannexin 1 has been postulated to mediate or facilitate passive transport of ATP across the plasma membrane of many cell types, including RBCs from humans and other vertebrate species [13]–[17]. During *P. falciparum* infection, on the other hand, new permeability pathways are activated, including the activation of anion channels [56], [31]. Thus, new potential ATP conduits might arise during infection, thereby altering the relative contribution of pannexin 1 to ATP release in infected RBCs.

Thus, conduits of ATP release were assessed by analyzing ATPe kinetics of RBCs from h- and t94-RBCs, both in the absence and presence of carbenoxolone, mefloquine or NPPB. Carbenoxolone and mefloquine are two well known blockers of pannexin 1 [20], [25], whereas NPPB was reported to inhibit ATP generated currents of *P. falciparum* parasitized RBCs [31] and to block pannexin 1 currents in a mammalian cell expression system [57].

In t94-RBCs 3V dependent ATPe accumulation was upregulated, as compared to h-RBCs. All inhibitors were able to inhibit ΔATP_1_ by 84 (h-RBCs) and 68% (t94-RBCs), thus highlighting the importance of pannexin-1 as the main ATP conduit of both cell types. Nevertheless, the lower relative inhibition of ΔATP_1_ in t94-RBCs, as compared to h-RBCs, suggests activation of a residual ATPe efflux. Besides pannexin 1, one or more transport proteins can in principle account for the observed CBX/MFQ/NPPB-tolerant component of ATP efflux of t-RBCs. In this respect, Sridharan et al. [58] identified a voltage dependent anion channel VDAC 1 in the plasma membrane of h-RBCs, and provided pharmacological evidence that prostacyclin receptor-mediated ATP release from erythrocytes can be blocked by inhibitors of this channel, but not by inhibitors of pannexin 1. More recently, another voltage gated anion channel CALHM1 was shown to mediate ATP exit from taste bud cells [59].

Up to now it is not known whether these postulated conduits mediating ATP release function as a single entities, or in physical association with other proteins e.g. cytoskeleton components and purinergic receptors. In neurons, pannexin 1 associates in a multiprotein inflammasome complex including P2X7 receptor and caspase 1 [19].

To further check the effect of *P. falciparum* infection on ATPe regulation, ATPe kinetics of h- and t94-RBCs was analyzed when cells were exposed to mastoparan 7 (MST7). We wanted to know whether the observed changes in ATPe kinetics brought about by *P. falciparum* infection could also be triggered by a stimulus chemically unrelated to 3V. The peptide MST7 triggers cAMP-dependent ATP release of h-RBCs by activating specific adenylyl cyclases isoforms, and other signalling factors (see [60], [25]). As shown in Fig. S4A, MST7 exposure of h-RBCs led to an acute nonlinear increase of [ATPe], corroborating previous findings [25]. Similarly to 3V exposure, ATP release of MST7 stimulated t94-RBCs was highly activated, so that ΔATP1 was 6.4-fold higher than in h-RBCs exposed to the same treatment. Thus, in an almost pure population of trophozoites (t94-RBCs) two qualitatively different stimuli induced a 4–6 -fold activation of ATP efflux.

### Modulators of ATP Release: ATPi, ATPe and Nitric Oxide (NO)

The analysis above suggests that, following 3V mediated activation, h-RBCs and t-RBCs both use pannexin-1 as the main conduit of 3V dependent ATP release, and that ATP efflux is enhanced as a consequence of infection. Several factors may act simultaneously to modulate ATP efflux.

#### Intracellular ATP

We have recently reported that, in h-RBCs, ATP efflux depends mainly on ATP permeability (a kinetic factor) and intracellular ATP (a thermodynamic factor), with almost no effect exerted by ATPe [25]. Therefore, if all other factors affecting ATP efflux remained similar in h-RBCs and t-RBCs, the 45% lower cytosolic ATPi concentration found in t94-RBCs (as compared to h-RBCs, Fig. S1A) should have led to a lower ATP efflux in t-RBCs. Thus, the fact that ATP efflux was enhanced as a consequence of infection suggests that enhancements of ATP permeability of t-RBCs highly surpass the inhibitory effect of a diminished [ATPi].

#### Nitric oxide

Endothelial vascular cells can not only release NO abluminally to promote vasodilation, but they can also release NO in the vascular lumen, where it can inhibit ATP release from h-RBCs [10]. On the other hand, Rathathagala et al. [61] showed that low NO concentrations activate, and high NO concentrations inhibit ATP release of rabbit RBCs. Irrespective of the effect of endothelial derived NO, we aimed to analyze if intraerythrocytic NO affects ATP release of RBCs. Results indicate that, in t-RBCs, extracellular NO_2_ concentration increased with parasitemia, pointing to an enhanced NO production induced by infection. Accordingly, intracellular NO production in t94-RBCs was much higher than in h-RBCs, and it was located near the parasite. Inhibition of NO production with L-NAME suggests the presence of an active NO synthase in parasitized cells. This agrees well with the fact that *P. falciparum* expresses a L-NAME-sensitive NO synthase isoform capable of NO synthesis [62], while hemoglobin concentration (a major NO sink under normoxic conditions) is highly reduced (as compared to h-RBCs) [43]. On the contrary, although h-RBCs express an eNOS-like protein capable of NO synthesis, its functional significance is controversial, and the high intraerythrocytic hemoglobin concentration would act as a major sink of the intracellularly generated NO, thus explaining the relative low NO production observed in these cells.

Given that NO production is enhanced in t94-RBCs, we investigated the effects of L-NAME on ATP efflux. Both in h-RBCs and t94-RBCs, L-NAME increased 3V-dependent ATP release, with the effects on t94-RBCs being stronger.

Interestingly, the effect of L-NAME on ATP release of t-RBCs is observed over a wide range of parasitemias, stressing the modulatory role of NO on ATP release.

As evidenced from the inhibition with CBX, enhancement of ATP release in t-RBCs requires the activation of pannexin 1.

A clue to the underlying mechanism can be obtained from results showing an enhanced ATP release by L-NAME exposure of t-RBCs. It is known that NO inhibits eryptosis (apoptosis of anucleated erythrocytes) in *P. falciparum* infected h-RBCs, an effect significantly more marked than in noninfected RBCs [63], [64]. In Jurkat T cells, on the other hand, caspase 3 activation during apoptosis results in the cleavage of a specific segment of pannexin 1, leading to its activation [65]. Thus, it is possible to speculate that in t-RBCs under L-NAME exposure, inhibition of NO production would trigger eryptotic caspase activation followed by activation of pannexin 1.

### Physiological Significance

The quantitative ATPe profile described for human RBCs in the present and in two previous studies [66], [12] is compatible with an *in vivo* scenario where, under nonstimulated conditions, [ATPe] of h-RBCs is maintained constant at a relatively low value, and acute increases occur in response to certain physiological and/or pathological conditions. During malaria, two important factors would alter ATPe homeostasis. First, plasma ATPe concentrations are elevated in malaria patients [55]. This has been usually associated with a high degree of hemolysis found *in vivo* [67]. However, after 24 hs culture of t-RBCs (at 4–5% parasitemia) we found 100-fold higher [ATPe] concentrations than in h-RBCs, while no hemolysis was detected. This means that basal ATPe efflux is more elevated in infected cells, a feature compatible with our analysis of ATPe kinetics. Second, in 3V stimulated cells there is a time-dependent enhancement of [ATPe] caused by *P. falciparum* infection. This can be relevant *in vivo,* where levels of cathecolamines and other ATP stimulating factors can be high [68].

**Figure 10:**
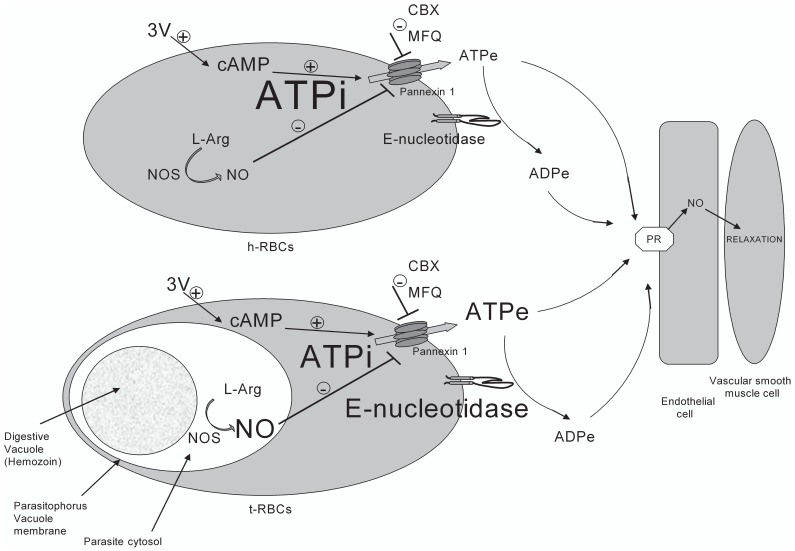
Qualitative scheme depicting main differences in 3V-induced ATPe regulation of noninfected and trophozoite-infected RBCs (h- and t-RBCs) and the consequences of ATPe and ADPe accumulation on endothelial mediated vasodilation. Trophozoite-infected RBCs (t-RBCs) produce nitric oxide (NO) at the site where the parasite is located. A comparison of the responses in both cell types show that time dependent ATPe concentrations are higher in t-RBCs than in h-RBCs, despite higher inhibition of ATP efflux by a relative high NO production, and high ATPe degradation rate by ectoATPase activity.

Moreover, this elevated [ATPe] occurred in t-RBCs over a wide range of parasitemias, and even in the presence of a diminished ATPi concentration (decreasing the driving force for ATP efflux), a strong enhancement of ectoATPase activity (promoting ATPe degradation) and an enhanced production of NO (inhibiting ATP release) (see scheme of Fig. 10). Thus, provided that in particular areas of the circulation the rates of ATPe hydrolysis by different ecto- and exo-nucleotidases of blood cells, the vascular endothelium and plasma are not high enough, and that paracrine distances from ATP exit are short, an elevated [ATPe] induced by infected cells may activate P2 receptors of the vascular endothelium to enhance vasodilation.

What about ATPe homeostasis of mefloquine treated patients? Mefloquine, a synthetic analogue of quinine, is used to prevent and treat malaria, particularly in areas where *P. falciparum* is resistant to chloroquine [69]. Our results indicate that 100 nM mefloquine inhibited 89–90% of 3V-induced ATP release from both h- and t94-RBCs (Fig. 4B). This concentration is about 200–600 times lower than that found in plasma of malaria patients after 48 hs of receiving a single 1 g-dose [70]. This means that during mefloquine-treated malaria, even at low parasitemia, activation of ATP efflux of h-and t-RBCs would be highly compromised by this drug, with potential consequences on ATPe-dependent vasodilation.

Since RBCs derived ATPe was also shown to modulate parasite invasion [71], future studies could help to determine how important is ATPe homeostasis generated by infected RBCs as a target for antimalarial treatments.

## Supporting Information

Figure S1 **Cytosolic ATP content (ATPi) of h- and t94-RBCs and extracellular ATP (ATPe) of h- and t5-RBCs cultures.** A. The cytosolic ATP content of trophozoite-infected RBCs (t-RBCs) and noninfected RBCs (h-RBCs) was determined by luciferase-luciferin luminometry after permeabilization of cells with digitonin (50 µg/ml) (see Materials and Methods). By considering the cytosolic volume of these cells [43], the intracellular ATP concentration could be derived. Results are means ± SEM (N = 5, n = 5). (**p<0.01). B. ATPe content was determined in noninfected RBCs (h-RBCs) and trophozoite-infected RBCs at 5% parasitemia (t5-RBCs) cultures. Cells were cultured 24 hs at 5% hematocrit in supplemented RPMI medium at 37°C. Suspensions were centrifuged 3 min at 900 *g* and an aliquot of the supernatant was used for an off-line determination of ATPe (see Materials and Methods). Results are means ± SEM (N = 3, n = 3). (***p<0.001).
(TIF)

Figure S2 **Kinetics of viability of h-RBCs and t94-RBCs.** Viability (as %) was assayed continuously in BCECF-loaded RBCs by fluorescence microscopy in the absence and presence of 3V. The assay chamber used for these experiments was similar to that used for luminometry experiments. By repeating this procedure for 3–5 independent preparations we found that: In noninfected RBCs (h-RBCs) one cell died (out of 141) at 23 min post-stimulus. In trophozoite-infected RBCs at 94% parasitemia (t94-RBCs) one cell died (out of 253) in the pre-stimulus phase, and another cell died at 26 min post-stimulus.
(TIF)

Figure S3 **ATPe] kinetics of mice t-RBCs infected with** ***P. chabaudi*** **.** A. The time course of ATPe concentration ([ATPe]) was assessed for mice RBCs (m-RBCs) (CF-1 strain) and quantified by real-time luminometry, as described in Materials and Methods for h-RBCs. In the time indicated by the arrow, cells were exposed to “3V”, a cAMP activating cocktail containing 10 mM isoproterenol, 30 mM forskolin and 100 mM papaverine. Levels of ATPe were expressed both as pmol ATP/(10^6^ cells) (left axis) or as ATPe concentration (nM) with 10^6^ cells in 60 µl assay volume (right axis). Data represent mean values ± SEM from N = 2 independent preparations. B. The time course of [ATPe] (pmol/10^6^ cells) was assessed for *P. chabaudi* infected mice (CF-1 strain) RBCs, at trophozoite stage and ≈80% parasitemia (denoted as t80-mRBCs). Experiments were run in the absence and presence of 100 µM carbenoxolone (CBX) in 2 independent preparations. C: For a comparison, ATPe kinetics of trophozoite-infected RBCs at 94% parasitemia (t94-RBCs) taken from Fig. 4A is shown. Exposure to 3V is indicated by the arrow. D: The effect of pre-incubation with carbenoxolone for 10 min before the exposure to “3V” was evaluated. The difference between [ATPe] at 1 min post-stimulus and the basal [ATPe], indicated as ΔATP_1_, was expressed as a percentage of the value obtained with no incubation with carbenoxolone for t80-mRBCs and t94-RBCs.
(TIF)

Figure S4 **MST7-dependent [ATPe] kinetics of h-RBCs and t94-RBCs infected with** ***P. falciparum*** **.** A. The time course of ATPe concentration ([ATPe]) was assessed for noninfected RBCs (h-RBCs) and trophozoite-infected erythrocytes at 94% parasitemia (denoted as t94-RBCs) and quantified by real-time luminometry, as described in Materials and Methods. In the time indicated by the arrow, cells were exposed 10 µM of mastoparan 7 (MST7). Data represent mean values from N = 2 independent preparations. B. MST7 and 3V-dependent increases of [ATPe] calculated from A (MST) and Fig. 4 (3V). Values are expressed as ΔATP_1_, i.e., the difference between [ATPe] at 1 min post-stimulus and basal [ATPe]. Results are means ± SEM. (*p<0.05, ***p<0.001). (N, n), with N = independent preparations, n = replicates.
(TIF)
