# Supplementary figures and images for: Regulation of Extracellular ATP in Human Erythrocytes Infected with Plasmodium falciparum
Source: PLoS One. 2014 May 23;9(5):e96216. doi: 10.1371/journal.pone.0096216 (PMC4032238; doi:10.1371/journal.pone.0096216)

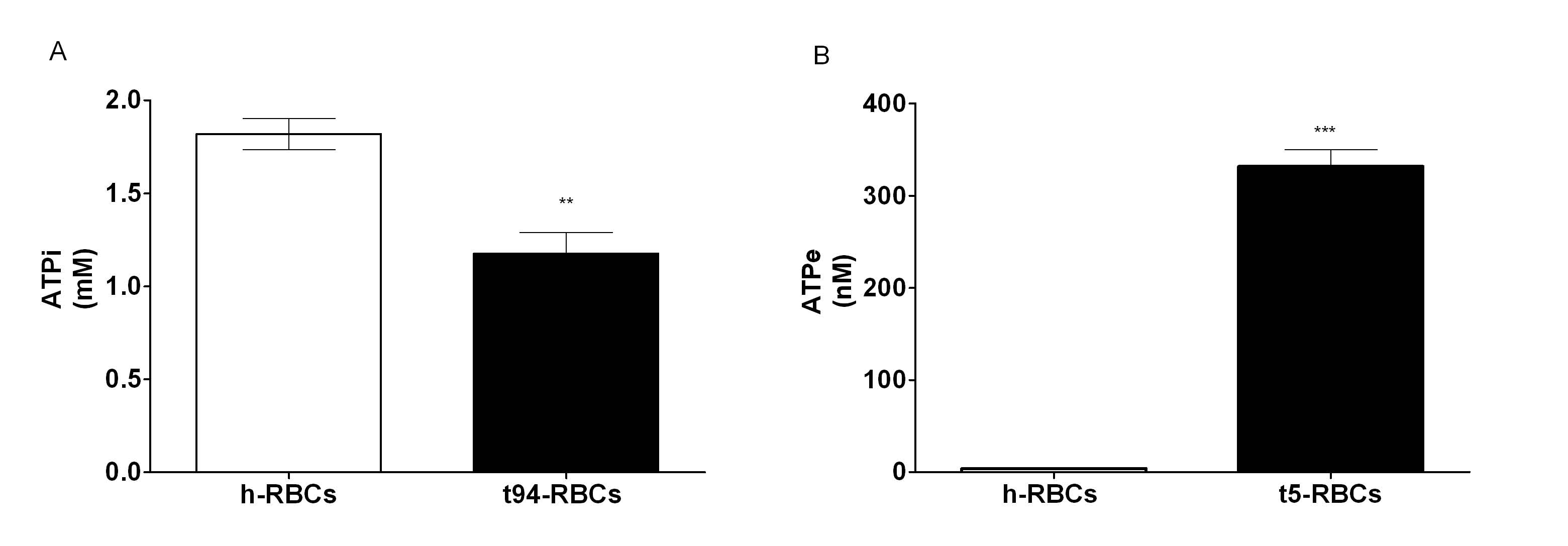

Supplement: Figure S1 — Cytosolic ATP content (ATPi) of h- and t94-RBCs and extracellular ATP (ATPe) of h- and t5-RBCs cultures. A. The cytosolic ATP content of trophozoite-infected RBCs (t-RBCs) and noninfected RBCs (h-RBCs) was determined by luciferase-luciferin luminometry after permeabilization of cells with digitonin (50 µg/ml) (see Materials and Methods). By considering the cytosolic volume of these cells [43], the intracellular ATP concentration could be derived. Results are means ± SEM (N = 5, n = 5). (**p<0.01). B. ATPe content was determined in noninfected RBCs (h-RBCs) and trophozoite-infected RBCs at 5% parasitemia (t5-RBCs) cultures. Cells were cultured 24 hs at 5% hematocrit in supplemented RPMI medium at 37°C. Suspensions were centrifuged 3 min at 900 g and an aliquot of the supernatant was used for an off-line determination of ATPe (see Materials and Methods). Results are means ± SEM (N = 3, n = 3). (***p<0.001). (TIF) [file pone.0096216.s001.tif]

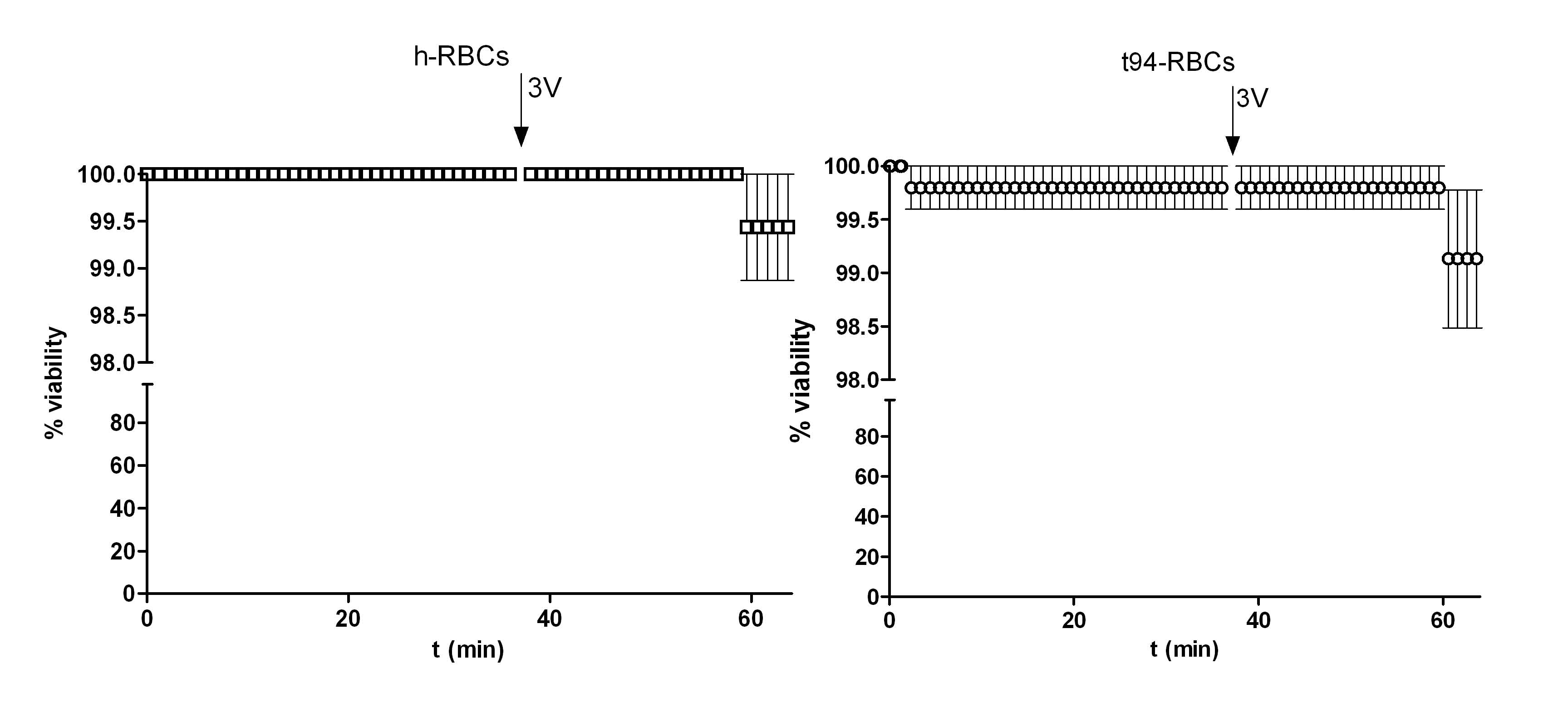

Supplement: Figure S2 — Kinetics of viability of h-RBCs and t94-RBCs. Viability (as %) was assayed continuously in BCECF-loaded RBCs by fluorescence microscopy in the absence and presence of 3V. The assay chamber used for these experiments was similar to that used for luminometry experiments. By repeating this procedure for 3–5 independent preparations we found that: In noninfected RBCs (h-RBCs) one cell died (out of 141) at 23 min post-stimulus. In trophozoite-infected RBCs at 94% parasitemia (t94-RBCs) one cell died (out of 253) in the pre-stimulus phase, and another cell died at 26 min post-stimulus. (TIF) [file pone.0096216.s002.tif]

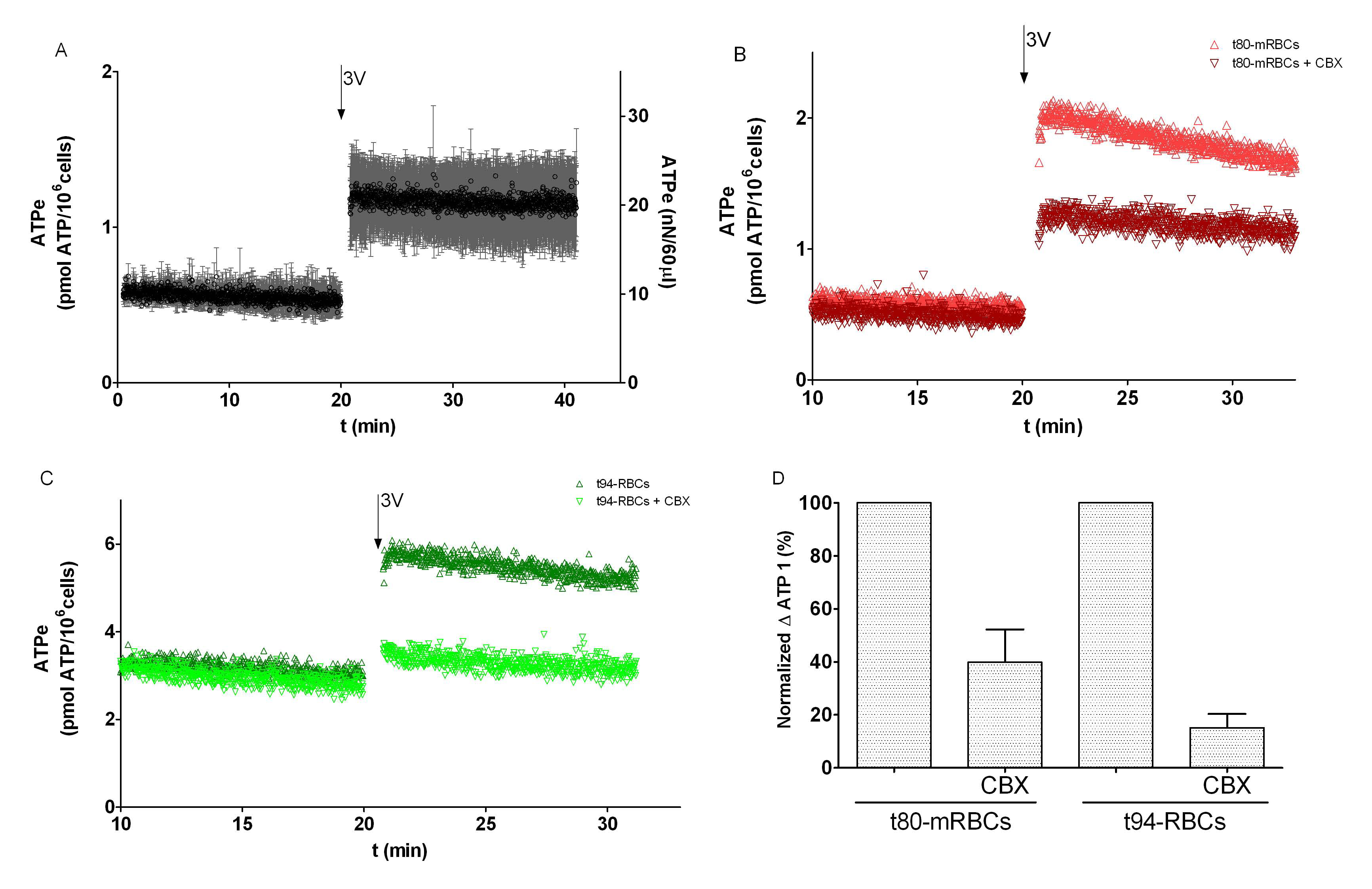

Supplement: Figure S3 — ATPe] kinetics of mice t-RBCs infected with P. chabaudi . A. The time course of ATPe concentration ([ATPe]) was assessed for mice RBCs (m-RBCs) (CF-1 strain) and quantified by real-time luminometry, as described in Materials and Methods for h-RBCs. In the time indicated by the arrow, cells were exposed to “3V”, a cAMP activating cocktail containing 10 mM isoproterenol, 30 mM forskolin and 100 mM papaverine. Levels of ATPe were expressed both as pmol ATP/(106 cells) (left axis) or as ATPe concentration (nM) with 106 cells in 60 µl assay volume (right axis). Data represent mean values ± SEM from N = 2 independent preparations. B. The time course of [ATPe] (pmol/106 cells) was assessed for P. chabaudi infected mice (CF-1 strain) RBCs, at trophozoite stage and ≈80% parasitemia (denoted as t80-mRBCs). Experiments were run in the absence and presence of 100 µM carbenoxolone (CBX) in 2 independent preparations. C: For a comparison, ATPe kinetics of trophozoite-infected RBCs at 94% parasitemia (t94-RBCs) taken from Fig. 4A is shown. Exposure to 3V is indicated by the arrow. D: The effect of pre-incubation with carbenoxolone for 10 min before the exposure to “3V” was evaluated. The difference between [ATPe] at 1 min post-stimulus and the basal [ATPe], indicated as ΔATP1, was expressed as a percentage of the value obtained with no incubation with carbenoxolone for t80-mRBCs and t94-RBCs. (TIF) [file pone.0096216.s003.tif]

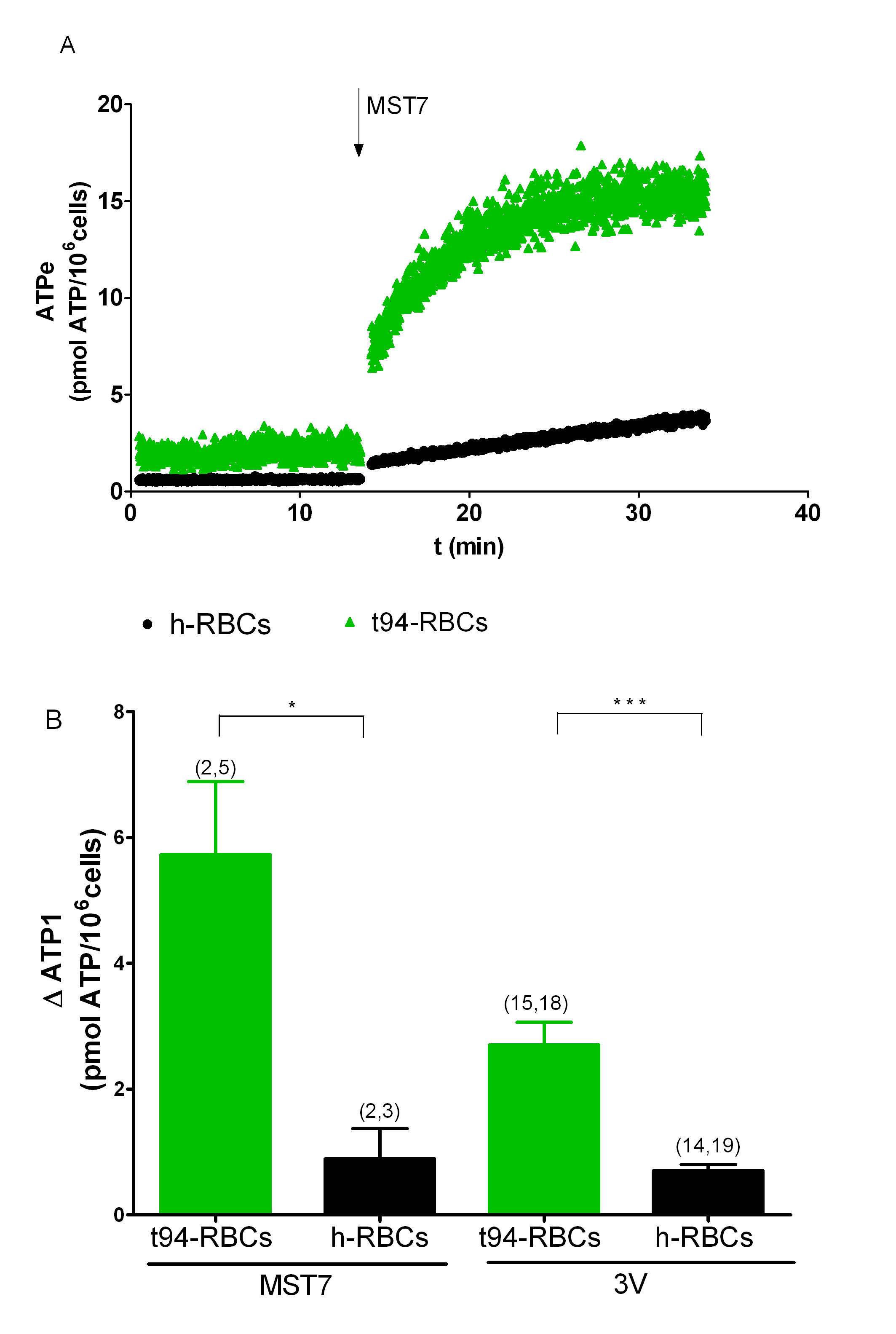

Supplement: Figure S4 — MST7-dependent [ATPe] kinetics of h-RBCs and t94-RBCs infected with P. falciparum . A. The time course of ATPe concentration ([ATPe]) was assessed for noninfected RBCs (h-RBCs) and trophozoite-infected erythrocytes at 94% parasitemia (denoted as t94-RBCs) and quantified by real-time luminometry, as described in Materials and Methods. In the time indicated by the arrow, cells were exposed 10 µM of mastoparan 7 (MST7). Data represent mean values from N = 2 independent preparations. B. MST7 and 3V-dependent increases of [ATPe] calculated from A (MST) and Fig. 4 (3V). Values are expressed as ΔATP1, i.e., the difference between [ATPe] at 1 min post-stimulus and basal [ATPe]. Results are means ± SEM. (*p<0.05, ***p<0.001). (N, n), with N = independent preparations, n = replicates. (TIF) [file pone.0096216.s004.tif]
